# Markers of Toxicity and Response to Radiation Therapy in Patients With Prostate Cancer

**DOI:** 10.1016/j.adro.2020.10.016

**Published:** 2020-10-27

**Authors:** Nicola J. Nasser, Jonathan Klein, Abed Agbarya

**Affiliations:** a Department of Radiation Oncology, Montefiore Medical Center and Albert Einstein College of Medicine, Bronx, New York; b Institute of Oncology, Bnai Zion Medical Center, Haifa, Israel

## Abstract

The main treatment modalities for localized prostate cancer are surgery and radiation. Surgery removes the whole prostate gland, whereas with radiation therapy the irradiated prostate remains within the patient's body. Biomarkers specific to the prostate gland should become undetectable after surgery, but this is not the case when radiation therapy is used, as residual prostate cells may still be metabolically active. Here, we review the role of tumor markers of toxicity and response to radiation therapy in patients with prostate cancer, including prostate specific antigen, human kallikrein 2, osteopontin, prostate cancer associated 3, citrulline, and others.

## Introduction

**Figure 1:**
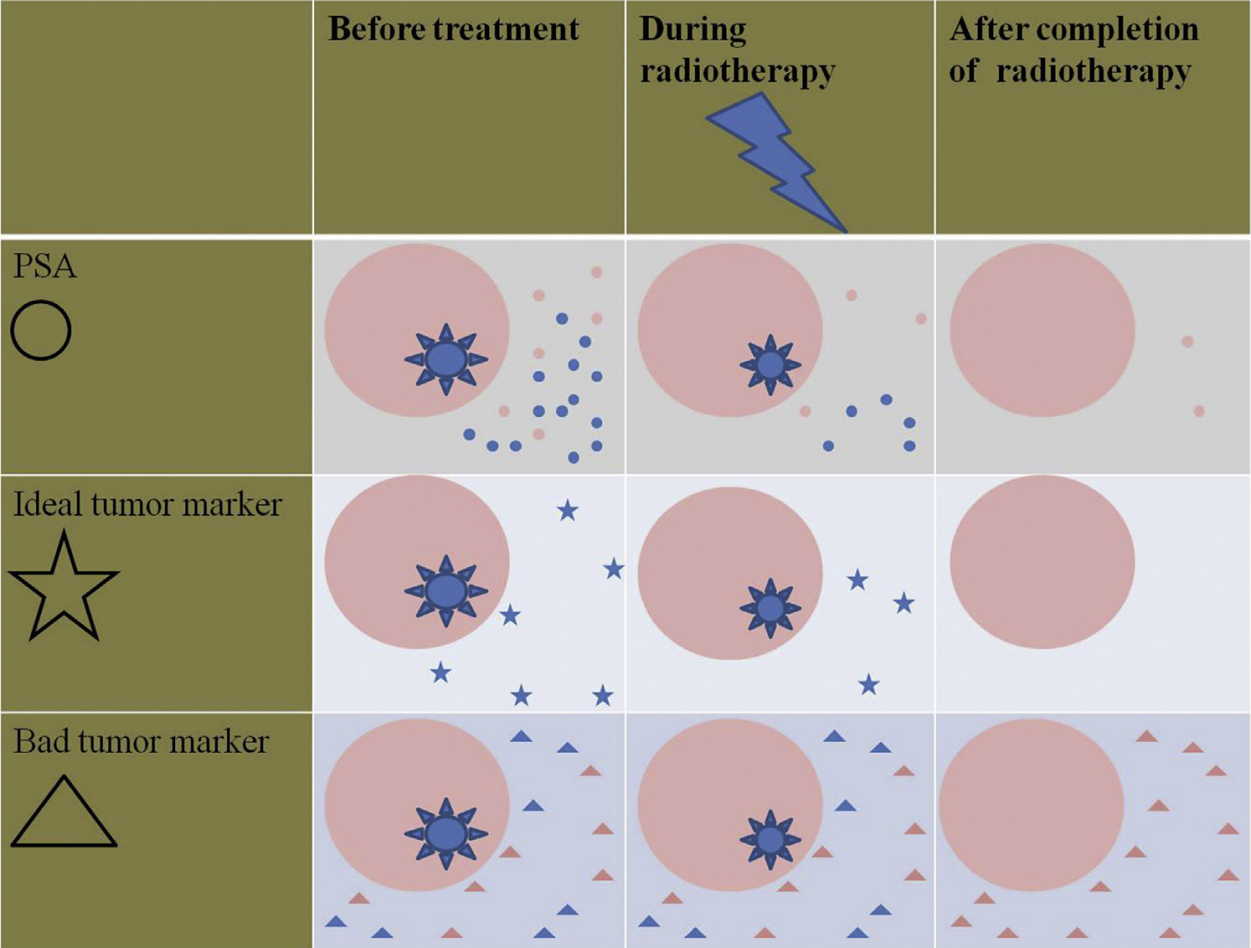
Tumor markers in patients with prostate cancer treated with radiation. Molecules secreted from nonmalignant prostate cells are in pink, and those secreted from prostate tumors are in blue. Prostate specific antigen (PSA) is secreted from both the normal prostate tissues as well as from tumor cells. After tumor ablation by radiation, PSA may still be secreted from irradiated nonmalignant prostate cells. Ideal tumor marker is a marker secreted by tumor cells, but not by normal prostate epithelium. The blood levels of the ideal tumor marker decrease as a function of the volume of tumor cell kill by radiation and become undetectable in blood once cure is attained. Bad tumor makers are those secreted from malignant and nonmalignant tumor cells and their levels are not much affected by radiation therapy.

Prostate cancer is the most prevalent malignancy in men, accounting for almost 1 in 5 new cancer diagnoses.^1^ The main treatment modalities for localized prostate cancer are surgery and radiation therapy (RT). Whereas surgery aims to remove the whole prostate and surrounding tissues (eg, seminal vesicles, peri-prostatic tissues, and pelvic lymph nodes), when RT is used, the irradiated prostate gland remains in situ within the patient's body. Therefore, biomarkers specific to the prostate gland (eg, prostate specific antigen (PSA), which reflects both normal and prostate cancer epithelium) should become undetectable after surgery, but this is not the case when RT is used, as residual prostate cells may still be metabolically active and produce markers. An ideal blood biomarker of response to RT in prostate cancer must accurately delineate between malignant and normal prostate epithelium (Fig 1). This ideal marker should: (1) be specific to prostate cancer rather than the prostate normal tissue; (2) decrease with RT in parallel with tumor cell kill; and therefore, (3) become undetectable when cure is achieved (Fig 1). Such an ideal biomarker has yet to be found.

Markers of radiation-induced late toxicity in normal tissues are also important. The vicinity of the anterior rectal wall^2^ and the bladder to the prostate results in their exposure to high doses of radiation when the prostate is treated. Whereas limiting radiation dose to normal structures helps to reduce toxicity, severe (Radiation Therapy Oncology Group [RTOG] grade 3 or higher) gastrointestinal (GI) or genitourinary (GU) late toxicities, defined as any radiation-related toxicity that occurs >180 days from the start of treatment,^3^ may develop in 2% to 6% of patients.^3^, ^4^, ^5^ Ideal surrogate blood biomarkers for late toxicity would be those that herald long-term effects early within the RT treatment when radiation-induced inflammation or injury to the rectum or bladder has not manifested clinical symptoms, and when permanent injury could potentially be prevented (ie, when toxicity is still reversible). This would allow for the identification of patients at risk for severe injury, and prompt reassessment of the RT plan for possible further reduction of dose to normal structures at risk.

## Blood Biomarkers of Prostate Cancer Radioresponse

The current review focuses on blood biomarkers of response to RT. Physical examination, clinically guided imaging, and biopsies of suspicious tumor recurrences may all provide information pertaining to macroscopic local recurrence. However, blood biomarkers (such as PSA) have the potential to declare recurrence after RT when the recurrence is subclinical or microscopic. We will review the utility and premise of PSA and other biomarkers in assessing response to RT for prostate cancer.

## PSA

PSA was introduced as a screening tool, and it is now used also as a marker of response to therapy. The first assay to test serum PSA was described by Kuriyama et al^6^ in 1980. PSA is a kallikrein serine protease expressed at high levels in prostate epithelium.^7^^,^^8^ PSA is not specific to prostate, and low levels have been reported in some women.^7^^,^^8^ In postmenopausal women, serum PSA detection has been shown to be significantly higher in patients with breast cancer compared with controls.^9^ An increase in PSA in men occurs naturally with increasing age, due to increasing prostatic volume associated with benign prostatic hyperplasia^10^ and after ejaculation.^11^ PSA can also be increased in nonmalignant conditions such as prostate infection resulting in acute^12^ or chronic^13^ prostatitis, or due to prostatic trauma.^14^ PSA as a biomarker of response to RT necessitates first the exclusion of nonmalignant causes as contributing to the high PSA levels.

During RT, the planning target volume is defined as the entire prostate plus a margin of 3 to 10 millimeters beyond the prostatic capsule.^15^^,^^16^ However, because of these margins, RT necessarily treats malignant in addition to normal prostate cells. Thus, PSA secreted from tumor as well as from a benign gland will decrease as a function of increasing RT dose. Moreover, androgen deprivation therapy (ADT), provided to intermediate^17^, ^18^, ^19^, ^20^ and high-risk^21^, ^22^, ^23^, ^24^ patients with prostate cancer before, during, or after RT further suppresses PSA secretion. PSA decreases during and after external beam RT and reaches nadir about 15 months after RT completion.^25^ It is thought that the length of time to reach a nadir PSA value after RT reflects cell kill due to cancer cell permanent growth arrest and mitotic catastrophe, which occurs months after the original RT dose.^26^ An increase of PSA after RT may be due to restoration of testosterone secretion, benign prostatic tissue with PSA secreting ability, or tumor recurrence. Nejat et al^27^ found that median time to normalization of testosterone after withdrawal of ADT was 7 months, with significantly longer time to return to normal in patients on ADT for 2 years or more. The complexity introduced by PSA recurrence led to development of guidelines (outlined in the following sections) that are designed to help clinicians to discriminate between benign conditions, in which no further investigation or treatment should be provided, and tumor recurrences necessitating restaging and further treatment consideration.

### PSA in response to external beam radiation therapy (EBRT)

#### American Society for Therapeutic Radiology and Oncology (ASTRO) consensus definition

In 1997, ASTRO published an expert consensus definition of PSA failure after RT, termed *biochemical failure*.^28^ According to the consensus, serum PSA should be measured every 3 to 4 months in the first 2 years after RT, and every 6 months thereafter.^28^ Biochemical failure, according to the ASTRO definition, was defined as 3 consecutive PSA rises after a nadir, with the date of failure defined as the point halfway between the nadir date and the first rise, or any rise great enough to provoke initiation of salvage therapy.^28^ Biochemical failure is not equivalent to clinical failure and is not justification per se to initiate additional treatment.^28^ Backdating of PSA failure using the ASTRO definition intended to account for more accurate timing of the PSA failure, although it also introduced a bias in reporting clinical trials, as series with short follow-up times could have better biochemical free survival (BFS) rates compared with studies with longer follow-up time.

#### Phoenix definition of PSA failure

In 2005, a conference sponsored by ASTRO and the RTOG was held in Phoenix, Arizona, to redefine PSA failure after EBRT.^29^ According to the panel, PSA/biochemical success for an individual patient should be guided by clinical judgment. The Phoenix definition was intended to define biochemical failure for a population rather than for an individual. This “Phoenix definition” of PSA failure after RT is a rise in PSA by 2 ng/mL or more above nadir PSA.^29^ This excludes rises due to other nonmalignant conditions such as PSA bounce,^30^ receptive anal intercourse,^31^^,^^32^ prostatic trauma,^33^ prostatitis,^34^ or confirmed laboratory errors.^35^ Moreover, time of PSA failure was determined at PSA result time and not backdated as in the ASTRO definition. Investigators may continue to use the ASTRO definition of PSA failure for EBRT or brachytherapy, when no hormonal therapy was provided to the patient in addition.^29^

#### PSA nadir

Time to nadir PSA after prostate cancer treatment differs according to treatment modality. Because the biological half-life of PSA is 2 to 3 days,^36^ serum PSA levels should be undetectable within 6 weeks after radical prostatectomy.^37^ Treatment with RT works with a slow process of cell kill, producing gradual decrease in PSA readings to an eventual nadir in 1 to 5 years,^38^ with an average time to PSA nadir of 15 months.^25^ Patients with a lower absolute nadir value and longer time to nadir PSA after EBRT may have better prognosis.^39^^,^^40^ Pike et al^41^ recently reported that among men with localized disease treated with RT who did not achieve PSA level < 0.2 ng/mL, time to nadir PSA of less than 12 months was significantly associated with an increased prostate cancer specific mortality compared with patients with time to nadir PSA ≥ 12 months; this association was not observed among men with a PSA nadir of < 0.2 ng/mL.^41^

#### PSA bounce after EBRT

Feigenberg et al^42^ reported PSA bounce after EBRT in 27% of patients, with median magnitude and time to bounce of 0.6 ng/mL (range, 0.1-16.6) and 31 months (range, 4-60), respectively. PSA bounce >1.4 ng/mL was an independent predictor of freedom from biochemical failure, freedom from distant metastases, and increased cause-specific survival.^42^ Rosser et al^43^ reported PSA bounce, defined as an initial increase in serum PSA of at least 0.5 ng/mL followed by a decrease to prebounce baseline serum PSA values, in 12% of patients treated with EBRT. Mean time to PSA bounce was 9 months from the time of therapy. Respectively, the 1- and 5-year biochemical disease-free survival rates were 100% and 82.1% for patients with PSA bounce and 93.9% and 57.7% for those without PSA bounce.^43^

#### PSA doubling time (PSADT) after EBRT

PSADT is an important prognostic factor for patients with biochemical failure after RT. Patients with short PSADT have worse prognosis compared with patients with long PSADT. D'Amico et al^44^ showed that a posttreatment PSADT of less than 3 months and the specific value of the posttreatment PSADT when it is 3 months or more appear to be surrogate endpoints for prostate cancer–specific mortality (PCSM) after surgery or RT. Klayton et al^45^ reported the results of 423 patients with T1-3N0M0 prostate cancer who were treated with EBRT and experienced PSA failure after treatment. PSADT of more than 6 months, compared with patients with PSADT of less than 6 months, fared better in terms of freedom from distant metastasis, PCSM, and overall survival at 7 years after treatment.^45^ Lee et al^46^ reported that in men with nonmetastatic prostate cancer treated with combined hormonal therapy and RT, a posttreatment PSADT of 8 months or less was associated with worse clinical outcomes.

#### Interval to PSA failure

The prognosis of men with localized prostate cancer who were treated with RT and experience biochemical failure varies widely. Buyyounouski et al^47^ identified 1722 men with biochemical failure (using the Phoenix definition) from 3 separate institutions and found that an interval from end of RT to biochemical failure (IBF) of less than 18 months was associated with higher PCSM, compared with patients with IBF longer than 18 months. The 5-year cumulative incidence of PCSM for patients with IBF more than 18 months was 9.4% compared with 26.3% for patients with IBF of 18 months or less.^47^ Shilkrut et al^48^ retrospectively measured the IBF of high-risk patients with prostate cancer treated with EBRT who had biochemical failure. An IBF ≤ 18 months was associated with increased risk of distant metastasis and PCSM.^48^ Johnson et al^49^ showed that IBF can be useful as well after salvage RT. After salvage RT, IBF ≤ 18 months was associated with increased risk of distant metastasis, PCSM, and overall mortality.^49^

### PSA in response to brachytherapy

#### Low dose rate (LDR) brachytherapy

LDR brachytherapy provides continuous RT, albeit with decreasing intensity as a function of time and seed radiation half-life.^50^, ^51^, ^52^, ^53^ Cell kill by LDR brachytherapy is gradual, and cells destined to die due to RT may still produce PSA for years. PSA readings gradually decrease after LDR brachytherapy to an eventual nadir in 2 to 5 years,^38^ with an average time to PSA nadir of 3 to 4 years.^54^^,^^55^ The absolute level of nadir PSA may be prognostic for disease control, as patients attaining nadir PSA ≤ 0.35 ng/mL have been shown to be significantly more likely to experience long-term freedom from biochemical failure than those with higher nadir PSA values.^54^

Zelefsky et al^55^ reported the results of 310 patients treated with brachytherapy alone (without ADT) at Memorial Sloan Kettering Cancer Center. Among these patients, the median nadir PSA value was 0.1 ng/mL compared with 0.6 ng/mL among 192 patients treated with EBRT alone (*P* < .0001). Time to reach nadir was 43 months in the brachytherapy group compared with 23.5 months in EBRT patients (*P* < .0001).^55^

Crook et al^56^ reported data from 7 institutions for 14,220 patients with localized prostate cancer treated with LDR brachytherapy as single or combined modality. For patients with 4-year PSA ≤ 0.2 ng/mL, the freedom-from-recurrence rates were 98.7% at 10 years and 96.1% at 15 years.^56^

The definition of PSA bounce differs significantly between studies.^57^ Patients treated with LDR brachytherapy experience benign PSA bounces in about 40% to 50% of cases.^38^^,^^58^ This bounce is possibly due to a wave of synchronous prostatic cell death months after treatment. In one study, the median time to onset of PSA bounce was 15 months, and its median magnitude was 0.76 ng/mL.^38^ Merrick et al^59^ reported that PSA bounces tend to occur 6.5-59.9 months after brachytherapy, with a mean and median of 19.5 and 16.3 months, respectively. Crook et al^38^ reported PSA bounce of >2 ng/mL in 15% of patients. Younger patients have higher rates of PSA bounce.^38^^,^^58^ Patients who experienced a PSA bounce were less likely to have a biochemical failure.^58^

Lo et al^60^ found that PSA levels at 4 to 5 years after LDR prostate brachytherapy are a strong predictor of disease-free survival. Patients with PSA ≤ 0.4 ng/mL 48 months after LDR brachytherapy had <1% risk of disease relapse at 8 years, whereas all patients with PSA > 1.0 ng/mL at 48 months relapsed.^60^

#### High dose rate (HDR) brachytherapy

Yoshioka et al^61^ reported the PSA response to HDR brachytherapy delivered to 112 patients (15 low risk, 29 intermediate risk, and 68 high-risk patients) with prostate cancer. The prescribed dose was 54 Gy delivered in 9 fractions over 5 days. Of the 112 patients, 94 also received hormonal therapy. Five-year PSA failure-free rates for low-, intermediate-, and high-risk patients were 85%, 93%, and 79%, respectively. Significant prognostic factors for PSA failure were the initial PSA level and younger age. A subsequent dose reduction trial from the same group provided HDR brachytherapy for intermediate- and high-risk patients with prostate cancer, delivering 45.5 Gy in 7 fractions over 4 days.^62^ Three-year PSA failure-free rates for intermediate- and high-risk patients were 96% and 90%,^62^ suggesting that high rates of biochemical control are achievable with this lower HDR dose regimen.

Morton et al^63^ reported the results of Sunnybrook Odette Cancer Center of HDR monotherapy for low- and intermediate-risk prostate cancer using 2 fractions of 13.5 Gy delivered 1 week apart. Median PSA at 5-years posttreatment was 0.16 ng/mL, and BFS was 95%. The same group reported results from a combined modality treatment regimen for intermediate-risk patients with prostate cancer, providing a single dose of 15 Gy to the prostate using HDR, followed by EBRT providing 37.5 Gy in 15 fractions to the prostate and seminal vesicles. Five-year BFS with this regimen was 97.4%.^64^

Mehta et al^65^ from the University of California, Los Angeles, reported PSA bounce in 67 out of 157 patients (43%) after HDR monotherapy. Median magnitude of PSA bounce was 0.7 ng/mL, and its median duration was 9 months. Patients younger than 55 years had a statistically significant (*P* = .001) higher likelihood of experiencing a bounce, with odds ratio of 2.22. There was also a statistically significant higher probability of experiencing a PSA bounce for every unit decrease in Gleason score.^65^

Astorm et al^66^ from the University of Uppsala reported that PSA bounce after HDR brachytherapy and EBRT was associated with a lower risk for PSA failure. PSA bounce occurred in 26% of the patients, where 9% had a bounce amplitude > 2 ng/mL. Median time to bounce peak was 15 months with a median bounce value of 1.5 ng/mL.^66^

### Other potential proteins/tests

#### Human Kallikrein 2 (hK2)

Human Kallikrein 2 (hK2) is a prostate-specific kallikrein.^67^^,^^68^ Thus, after radical prostatectomy hK2 levels should become undetectable.^68^ Serum hK2 levels are usually about 10% (range, 1-20%) of serum PSA levels. hK2 increases during prostate cancer progression. Serum hK2 levels, used in addition to PSA, have been shown to enhance discrimination between patients with benign prostate disease and those with prostate cancer.^69^ Among 324 men prescreened with PSA for prostate cancer and referred for biopsy, patients with hK2 measurements within the highest quartile had a 5- to 8-fold increase in risk for prostate cancer.^70^ Mabjeesh et al^71^ showed that detection of mRNA of PSA and hK2 transcripts by polymerase chain reaction (PCR) in the peripheral blood of patients with prostate cancer during brachytherapy can predict the biochemical outcome. Patients with concurrent positive PSA and hK2 PCR results during brachytherapy had higher postoperative blood PSA values and a slower decline rate of PSA compared with patients with negative PSA and hK2 PCR results.^71^

#### Four kallikrein markers panel

The 4 kallikrein markers panel (4KLK) is composed of total PSA, free PSA, intact PSA, and hK2.^72^ Carlsson et al^72^ showed that the 4KLK was able to distinguish between low-risk prostate cancer and more aggressive disease after radical prostatectomy with good accuracy. A meta-analysis of the 4KLK included 8500 patients, of whom 2780 had prostate cancer.^73^ The 4 kallikrein panel demonstrated 8% to 10% improvement in predictive accuracy of prostate cancer with either the Base Clinical Model or the Base Laboratory Model for identifying any cancer or high-grade prostate tumors.^73^ The authors conclude that 48% to 56% of current prostate biopsies could be avoided by using this panel.^73^ Men with a PSA level of 3 ng/mL or more but defined as low-risk by the 4KLK were unlikely to develop metastatic prostate cancer.^74^ A recent study retrospectively measured 4KLK in cryopreserved blood collected at the ages of 50 or 60 years from unscreened, prostate-cancer free men.^75^ The cohort included 43,692 men from Sweden, with 2420 men diagnosed with prostate cancer during 20 years of follow-up. Nearly three-quarters of prostate cancer deaths occurred in men with PSA in the top quartile (>1.3 ng/mL in men 50 years old, or PSA > 2.3 ng/mL in men 60 years old at time of blood sample).^75^ Among men with moderately elevated PSA (≥2.0 ng/mL), the 4KLK improved discrimination of men with higher chances to develop prostate cancer compared with relying on the PSA test only.^75^ Men with elevated PSA but a low 4KLK score can be monitored with repeated blood 4KLK markers testing in place of immediate biopsy.^75^^,^^76^

Nasser et al^68^ have published results showing how blood 4 KLK protein levels change during and after treatment with EBRT for prostate cancer. These results show that serum hK2 and intact PSA levels decrease with RT faster than total and free PSA. Levels of hK2 and intact PSA decrease as fast as 2 weeks after initiation of RT, whereas the first significant decrease in total and free PSA is noted only at the completion of RT.^68^

The use of the 4KLK is still limited by the lack of availability of the test in many cancer centers. Although total and free PSA testing is available in many clinics, antibodies for intact PSA and hK2 used in most published series were custom made, and are not available in most cancer centers. Thus, the cost of these tests currently is high. Moreover, obtaining the absolute values of the 4KLK panel components does not easily result in calculating the risk of prostate cancer. The 4KLK parameter’s components are used in an algorithm that also includes age and digital rectal examination that estimates the probability of detecting prostate cancer via biopsy.^73^ Although the nomograms are available in published manuscripts,^74^ the lack of availability of a free computerized tool with easy input of panel component values and output of the probability of malignancy further limit the use of the 4KLK at this time. Recently, a commercially available test has been used and incorporated in a scientific publication.^75^

#### Kallikreins 6 and 11

Kallikrein 6 (KLK6) levels have been shown to be elevated in patients with multiple types of malignancies, including glioblastoma,^77^ colorectal,^78^ laryngeal,^79^ gastric,^80^ and ovarian cancers,^81^ as well as prostate cancer. Nasser et al^68^ investigated the role of KLK6 in prostate cancer. KLK6 was detected in patients after radical prostatectomy under biochemical control,^68^ indicating that the prostate is not the only organ that secretes this kallikrein. KLK6 blood levels in patients with intermediate-risk prostate cancer treated with definitive EBRT significantly decreased at 2 and 12 months following RT compared with the baseline levels before the initiation of RT, but not to undetectable levels.^68^ This indicates that KLK6 is secreted from the prostate gland and could potentially be a part of a panel of markers that tests the response to RT in patients with prostate cancer.

Kallikrein 11 (KLK11) expression in prostate cancer has an inverse association with tumor aggressiveness.^82^ KLK11 expression in gastric cancer appears to be associated with a better prognosis in patients treated with surgery and adjuvant chemoradiotherapy.^83^ Stronger KLK11 expression in non-small cell lung cancer appears to be associated with better survival rates.^84^

Nasser et al^68^ showed that in patients with intermediate-risk prostate cancer treated with definitive EBRT, KLK11 levels increased significantly during RT. They then return to levels similar to baseline around 8 weeks after the end of treatment, and continue to decrease at 12 months after completion of RT, eventually achieving levels below pretreatment baseline. The increase of KLK11 during RT may be due to acute cell injury releasing KLK11 into the blood. The decrease of KLK11 levels 1 year after the end of RT may be due to prostate and/or seminal vesicle cell death.

#### Osteopontin

Osteopontin is a small integrin-binding ligand N-linked glycoprotein that plays important roles in bone remodeling, immune response, and inflammation.^85^ High plasma osteopontin levels in patients with lung, breast, head and neck, and prostate cancers have been correlated with decreased disease-free and overall survival.^86^ Patients with metastatic castration resistant prostate cancer have increased baseline osteopontin values compared with patients with localized, nonmetastatic disease.^85^ Thoms et al^85^ reported that among patients with localized prostate cancer, osteopontin neither distinguished high-risk prostate cancer from other localized prostate cancer nor correlated with serum PSA at baseline. Another recent report found a positive correlation between osteopontin level and prostate cancer prostate clinical stage.^87^ Osteopontin levels were reduced after radical prostatectomy, but not always after EBRT despite reduced PSA levels.^85^ Interestingly, Wisniewski et al^88^ reported that osteopontin levels were significantly higher in patients with prostate cancer during and 1 month after RT compared with baseline. These data indicate that the role of osteopontin in monitoring localized prostate cancer following RT as a sole parameter of treatment efficacy is limited.

#### Prostate cancer associated 3 (PCA3)

PCA3, previously known as prostate cancer antigen 3 and as DD3, is a nonprotein coding RNA that is highly expressed in prostate cancer tumors.^89^ The PCA3 gene has been mapped to chromosome 9q21–22.^89^ PCA3 noncoding RNA is involved in the control of prostate cancer cell survival, in part through modulating androgen receptor signaling.^90^ PCA3 is measured in urine samples obtained after prostatic massage. The PROGENSA PCA3 Assay was approved recently by the United Sates Food and Drug Administration, and it measures the ratio between RNA copy number of PCA3 and PSA, multiplied by 1000.^91^ The cutoff value for a positive PCA3 test is controversial. A cutoff of PROGENSA PCA3 test of 35 resulted in sensitivity of 58% and specificity of 72% in diagnosing prostate cancer in men who had high PSA and a first negative biopsy.^92^ Prostate cancer was diagnosed in 35 out of 90 men presenting with urinary PCA3 scores ≥100, providing a positive predictive value of 38.9%.^93^ We do not know how PROGENSA score changes during and after RT. Measuring PCA3 during RT may not be feasible, as digital rectal prostate massage is needed for performing this test, and radiation proctitis may make such rectal massage intolerable for patients while undergoing RT. It will be interesting to see how PCA3 changes after RT (eg, 6-8 weeks and 1-2 years posttreatment) and whether this correlates with PSA or clinical responses.

#### Aberrantly expressed MicroRNAs (miRNAs) plasma levels

miRNAs are small noncoding RNA strands that function in regulation of gene expression.^94^ miRNA-375 and miRNA-141 expression is enhanced in prostate cancer specimens and their release into the blood is further associated with advanced cancer disease.^95^ Zidan et al^96^ reported that plasma levels of miRNAs -21, -125b, -126, -141, -143, and -375 were upregulated in patients with prostate cancer compared with individuals without malignancy. Mao et al^97^ reported that miRNA-141 in peripheral blood, measured with quantitative real-time PCR, was significantly higher in patients with prostate cancer compared with patients with benign prostatic hypertrophy.

Overall, blood miRNAs levels seem to be promising potential markers for prostate cancer that warrant further investigation. It will be also interesting to see how these markers respond to RT.

#### Heparanase

Heparanase is an endoglycosidase that degrades heparan sulfate on the cell surface and at the extracellular matrix.^98^^,^^99^ Heparanase is implicated in tumor growth, angiogenesis, and metastasis, and its expression is correlated with tumor aggressiveness.^98^^,^^100^^,^^101^ Nasser et al^101^, ^102^, ^103^, ^104^ cloned multiple splice variants of heparinase: one of them (splice #7) enhances tumor growth, and another (splice #36) functions as dominant negative to the wild type enzyme, suppressing extracellular matrix degradation, tumor growth, and metastasis.^101^^,^^105^ Moreover, heparanase enzyme secreted from the tumor cells degrades endogenous heparin, potentially contributing to the hypercoagulability in patients with cancer.^99^^,^^106^^,^^107^ Heparanase seems to play a role in early embryonic development and prostate morphogenesis.^108^ Malignant transformation in prostate cancer has been shown to be associated with considerable increase in the expression of heparanase at both mRNA and protein levels.^109^^,^^110^ Lerner et al^111^ reported a highly statistically significant (*P* < .0001) prevalence of heparanase overexpression in prostate carcinomas versus noncancerous tissue, as well as a strong correlation between tumor grade and the extent of heparanase expression.^111^ Whether heparanase may function as a biomarker of response to RT or if it will be able to discriminate between localized and metastatic disease is under investigation.

## Markers of Normal Tissue Toxicity in RT of Patients With Prostate Cancer

There are many disease conditions that could predispose patients to radiation toxicity. These include ataxia telangiectasia, which results from mutations in the ATM gene^112^; ataxia telangiectasia like disorder 1, which results from mutation in the MRE11 gene^113^; Nijmegen breakage syndrome, which results from mutations in the Nibrin (NBN) gene^113^; Seckel syndrome, which results from mutation of the ATR gene; Bloom syndrome caused by a mutation in the BLM gene^114^^,^^115^; Werner syndrome, which is caused by mutation in the WRN gene^116^; inflammatory bowel diseases^117^; collagen vascular diseases^118^; and others. These disease conditions are associated with hypersensitivity to RT and are beyond the scope of this review. We will focus on markers associated with toxicity in patients without known predisposing sensitivity to RT.

### Cytokine changes during RT and GU and GI toxicity

Christensen et al^119^ tested the association between cytokine expression and treatment toxicity during intensity modulated RT for prostate cancer. Increasing IL-2 and IL-1 expression was associated with increased probability of acute GI and GU toxicity, respectively.^119^ Kovacs et al^120^ demonstrated that IL-1α, macrophage colony-stimulating factor, and TGFβ blood levels increase during RT for the prostate and pelvis. Pinkawa et al^121^ found that early lymphocyte level elevation at 2 to 3 weeks after initiation of EBRT was protective against late urinary toxicity; decreasing ferritin levels and increasing TNFα levels 6 to 8 weeks after EBRT were found to be independent protective reactions against long-term bowel toxicity.^121^

### Plasma citrulline and intestinal toxicity

Plasma citrulline, a nitrogen end product of small bowel enterocyte glutamine metabolism, has been used as a marker for evaluating functional small bowel enterocyte mass.^122^ Because plasma citrulline concentrations are highly dependent on intestinal cell mass, low citrulline concentrations are associated with intestinal failure independent of underlying causes. Onal et al^122^ tested citrulline plasma levels in patients treated with pelvic RT for prostate or endometrial cancer. Decreases in citrulline concentrations were significantly correlated with intestinal toxicity during RT, at the end of radiation, and 4 months after completion of RT.^122^ Lutgens et al^123^ measured plasma citrulline in 23 patients treated with fractionated RT for abdominal or pelvic cancer sites, during and following completion of RT. Citrulline concentration significantly decreased as a function of the radiation dose. Maximal RTOG GI toxicity grade detected through the trial was 2. The plasma citrulline concentration correlated with clinical toxicity during the last 3 weeks of treatment, while no such relationship was observed during the first 3 weeks of treatment. Citrulline concentration correlated better with RT dose and volume parameters than clinical toxicity grading.^123^

### Fecal calprotectin and lactoferrin

Hille et al^124^ investigated 2 markers of gut inflammation, calprotectin and lactoferrin, during and after RT for prostate cancer. Calprotectin and lactoferrin values were significantly correlated with chronic proctitis, and all patients with chronic toxicity had acute proctitis symptoms with elevated fecal values. There are no long-term data with these markers for patients with prostate cancer undergoing modern era RT.

## Conclusions

At present, PSA remains the gold standard as the biomarker of choice for monitoring response to therapy in localized prostate cancer. A sustained and increasing PSA value following RT, in the absence of metastatic disease, will continue to be associated with posttreatment prostate imaging and biopsies. Novel biomarkers could potentially better discern the effects of RT on malignant versus normal tissues. This may be particularly important given a surge in focal therapy (eg, partial prostate volume treatment) in which the surrounding normal epithelium may not be ablated. The use of the 4KLK is still limited by the lack of accessibility to intact PSA and hK2 antibodies in many cancer centers. Research efforts should be focused on complimentary or new markers that are specific to prostate cancer cell death, to afford earlier intervention in the first 2 to 3 weeks of RT if the marker heralds local failure. Such patients could then undergo bioadaptive therapy with either dose modification or use of radiosensitizers to improve outcome.
